# Unmet Caregiving Needs Are Associated With Cognitive Functioning Among Older Sepsis Survivors

**DOI:** 10.1093/geroni/igaa057.820

**Published:** 2020-12-16

**Authors:** Jo-Ana Chase, Lizyeka Jordan, Christina Whitehouse, Kathryn Bowles

**Affiliations:** 1 University of Missouri, Columbia, Columbia, Missouri, United States; 2 Visiting Nurse Service of New York, New York, New York, United States; 3 Villanova University, Villanova, Pennsylvania, United States; 4 University of Pennsylvania School of Nursing, Philadelphia, Pennsylvania, United States

## Abstract

Sepsis survivorship is associated with cognitive decline and complex post-acute care needs. Family caregivers may be unprepared to manage these needs, resulting in decline or no improvement in patient outcomes. Using a national dataset of Medicare beneficiaries who were discharged from the hospital for sepsis and received post-acute HHC between 2013 and 2014 (n=165,228), we examined the relationship between unmet caregiving needs and improvement or decline in cognitive functioning. Multivariate logistic regression was used to determine associations between unmet caregiving needs at the start of HHC and changes in cognitive functioning. Unmet caregiving needs included seven items from the start of care Outcome and Assessment Information Set (OASIS). Changes in cognitive functioning were measured using the start of care and discharge OASIS assessments. Twenty-four percent of patients either declined or did not improve in cognitive functioning from HHC admission to discharge, with variation seen by unmet need type. Sepsis survivors with unmet caregiving needs for activities of daily living assistance (OR 1.05, 95% CI 1.01, 1.09), medication assistance (OR 1.06, 95% CI 1.02,1.10), and supervision and safety assistance (OR 1.110, 95% CI 1.06,1.16) were more likely to decline or not improve in cognitive functioning, even after accounting for clinical and demographic characteristics. Older sepsis survivors with both cognitive impairment and unmet caregiving needs in the post-acute HHC setting are at high-risk for worsening cognition. Alerting the care team of cognitively impaired sepsis survivors with unmet caregiving needs may trigger evidence-based strategies to enhance caregiver training and reduce unmet caregiving needs.

